# Cerebral blood flow response to dynamic resistance exercise

**DOI:** 10.1113/EP093313

**Published:** 2026-03-24

**Authors:** Stephanie Korad, Toby Mündel, Blake G. Perry

**Affiliations:** ^1^ School of Health Sciences Massey University Wellington New Zealand; ^2^ School of Sport, Exercise and Nutrition Massey University Palmerston North New Zealand; ^3^ Department of Kinesiology Brock University St Catharines Canada

**Keywords:** blood pressure, middle cerebral artery velocity, resistance exercise

## 1 INTRODUCTION

Connections link a sequence of three related research papers. The central article which links the other two papers has been published in *Experimental Physiology*. In a Connections article, an author (or authors) of the central article outlines its principal novel findings, tracing how they were influenced by the first article and how the central article has contributed to the developments made in the third article. The author(s) may also speculate on the direction of future research in the field. Connections articles aim to set the research in a wide context.

Few physiological stressors test the ability of the brain to regulate its blood flow as profoundly as dynamic resistance exercise (RE). Within seconds of commencing RE, blood pressure (BP) undergoes rhythmic oscillations that exceed the temporal buffering capacity of cerebral autoregulation, resulting in tightly coupled fluctuations in BP and cerebral blood flow (CBF). In addition to these rapid swings in BP, dynamic RE perturbs multiple regulators of CBF simultaneously, whether it be pre‐exercise hyperventilation and a reduction in arterial CO_2_, increased sympathetic drive and the associated cardiac responses, or motor cortex activation; the brain has a lot to deal with. Except for rowing exercise, this haemodynamic profile is unique to dynamic RE. In contrast, aerobic exercise, such as cycling, or even static RE (e.g. isometric handgrip contractions), elicit more gradual increases in BP that are readily accommodated by the cerebral circulation. However, the term ‘exercise’, despite being used frequently in the context of research, is non‐specific. Given that the acute cardiovascular and cerebrovascular responses are unique to the exercise type, an appropriate description of the type of exercise performed must be provided to avoid ambiguity.

What remains less well understood is whether repeated exposure to the haemodynamic stresses of habitual RE alters cerebrovascular regulation during subsequent bouts of dynamic RE. Specifically, does regular engagement in dynamic RE influence the capacity of the brain to stabilize perfusion during large BP oscillations during and immediately after RE? Furthermore, does the type of RE matter? Do different RE modes elicit different cerebrovascular responses? A series of recent articles published from a single comprehensive study addressed these research questions and, collectively, helped to refine our understanding of cerebrovascular regulation during dynamic RE. All data were collected as part of a single comprehensive study, enabling multiple research questions to be addressed using a unified dataset. Initially, we identified the pressure–flow dissociation during lower‐body dynamic RE, then examined whether this dissociation persisted during postexercise standing, and finally, tested whether similar CBF responses were observed across different RE modalities.

## 2 TRAINING STATUS REVEALS A PRESSURE–FLOW MISMATCH DURING LOWER‐BODY RE

In the first study of the series, we demonstrated that large differences in BP perturbations were not accompanied by proportional changes in middle cerebral artery (MCA) blood velocity (MCAv), a surrogate of CBF. In resistance‐trained individuals performing unilateral dynamic leg extensions, the magnitude of BP oscillations during RE was significantly greater than in untrained individuals, yet MCAv responses were remarkably similar between groups. This finding suggests that the relationship between arterial BP and MCAv differed between groups, with MCAv remaining stable despite larger BP perturbations in resistance‐trained individuals, and is consistent with previous observations. This raised an important physiological question: does this apparent pressure–flow dissociation persist once RE stops and acute reductions in arterial BP are imposed? The postexercise period represents a time of increased vulnerability to cerebral hypoperfusion, because rapid reductions in systemic BP during postural transitions can challenge MCAv regulation and contribute to symptoms such as dizziness or presyncope. Determining whether cerebral perfusion is maintained in these conditions provides important insight into the effectiveness of cerebrovascular buffering following dynamic RE.

## 3 DOES THE PRESSURE–FLOW MISMATCH PERSIST INTO RECOVERY AND ACUTE REDUCTIONS IN BP?

To determine whether the observed pressure–flow dissociation persisted beyond the exercise period, we examined cerebrovascular responses during the immediate recovery following dynamic RE. Specifically, we assessed cerebrovascular responses during postexercise standing to determine whether the pressure–flow dissociation observed during dynamic RE persisted during orthostatic stress. When participants stood up following dynamic RE, resistance‐trained individuals experienced larger reductions in BP than their untrained counterparts, a response that would conventionally be expected to compromise cerebral perfusion. However, MCAv reductions during postexercise standing were comparable between groups. Together, these findings indicate that habitual resistance training is associated with preserved MCAv stability despite exaggerated systemic BP perturbations, both during RE and during orthostatic stress immediately thereafter. Collectively, these findings suggest that MCAv was preserved despite rapid and substantial BP perturbations during dynamic RE. These observations prompted a crucial mechanistic question: how does the brain preserve blood flow stability in the face of extreme oscillatory pressure challenges? The stepwise study design narrows the likely contributors to those mechanisms capable of preserving MCAv across dynamic RE despite substantial differences in systemic BP.

**Connection Articles**

- Korad, S., Mündel, T., & Perry, B. G. (2024a). The effects of habitual resistance exercise training on cerebrovascular responses to lower‐body dynamic resistance exercise: A cross‐sectional study. Experimental Physiology, 109(9), 1478–1491.
- Korad, S., Mündel, T., & Perry, B. G. (2025a). Larger reductions in blood pressure during post‐exercise standing, but not middle cerebral artery blood velocity, in resistance‐trained versus untrained individuals. Experimental Physiology, 110(3), 424–437.
- Korad, S., Mündel, T., & Perry, B. G. (2025b). No difference in mean middle cerebral artery blood velocity responses between lower‐ and upper‐body unilateral resistance exercise in untrained individuals. Experimental Physiology, 1–17. https://doi.org/10.1113/EP092859

Several interacting mechanisms might contribute to the observed preservation of MCAv during dynamic RE. Dynamic cerebral autoregulation (dCA) is likely to play a central role by buffering fluctuations in cerebral perfusion pressure. However, autoregulatory effectiveness is strongly frequency dependent, with dCA most effective at attenuating slow BP oscillations (<0.05 Hz) and progressively less effective at higher frequencies (>0.10–0.20 Hz). During dynamic RE, rhythmic muscle contractions performed at ∼15 repetitions/min produce BP oscillations at ∼0.25 Hz, a frequency range known to challenge the temporal responsiveness of autoregulatory mechanisms. As a result, dCA remains active but is less able to buffer rapid pressure fluctuations fully, allowing partial transmission of oscillatory BP changes to MCAv. This is consistent with our findings, which demonstrate that MCAv varied with BP but to a lesser extent, indicating that autoregulatory mechanisms were engaged and limited pressure–flow coupling but did not eliminate it. However, without direct assessment of dCA, it remains unclear whether RE training alters autoregulatory function itself, which will require confirmation in longitudinal training‐intervention studies.

Thomas et al. (2021) used a cross‐over design to investigate the training‐associated vascular adaptations to 12 weeks of resistance or endurance exercise. They reported that resistance training increased cerebrovascular resistance at rest, whereas endurance training did not elicit comparable changes, suggesting that RE might induce resting cerebrovascular adaptations. Cross‐sectional work by Perry et al. (2019), using transfer function analysis of forced oscillations in BP induced by repeated squat–stand manoeuvres, demonstrated that dCA was largely preserved in resistance‐trained individuals, despite greater BP perturbations. However, a subsequent re‐analysis of these data by Roy et al. (2022) revealed altered directional sensitivity of the cerebral pressure–flow relationship in resistance‐trained individuals, with the absence of the hysteresis‐like pattern observed in sedentary and endurance‐trained individuals. These findings suggest that although autoregulatory buffering remains present, RE training might modulate the functional characteristics of cerebral autoregulation. Consistent with this interpretation, the present findings demonstrate preserved MCAv despite substantial oscillatory BP perturbations during dynamic RE and following acute postexercise orthostatic stress, indicating that autoregulatory mechanisms remain active in these conditions. However, longitudinal studies incorporating direct assessment of pressure–flow dynamics during RE will be required to determine whether resistance training alters autoregulatory function.

Neurovascular coupling (NVC) might contribute to the regulation of MCAv during dynamic RE. In our complementary work, we observed no differences between ipsilateral and contralateral MCAv responses during unilateral upper‐body dynamic RE (Korad et al., 2024b), despite asymmetric muscular activation. Although increased contralateral MCAv has been shown in handgrip exercise, and increased blood velocity is observed in the posterior cerebral artery during visual stimuli, the large concomitant perturbations in mean arterial pressure are likely to obfuscate the downstream NVC response in the MCA during dynamic RE. Given that NVC operates downstream of the MCA in the microvasculature of the motor cortex, it is likely that transcranial Doppler‐derived MCAv lacks the spatial resolution required to detect local neurovascular responses during dynamic RE, even if they are present.

An important contextual consideration is that all experimental conditions in the present studies were performed at a single, moderate intensity (60% of one‐repetition maximum) and involved a relatively small unilateral muscle mass. As such, the haemodynamic stimuli imposed during dynamic RE might differ from those experienced during higher‐intensity RE or bilateral large‐muscle‐mass movements that are commonly used in RE training. Future studies should therefore investigate cerebrovascular responses to higher‐intensity RE, engaging larger muscle masses and greater ranges of motion, to characterize the haemodynamic stimuli imposed by RE better.

## 4 DOES EXERCISE MODALITY (UPPER VS. LOWER BODY) MODIFY MCAv RESPONSES?

Because these comparisons were confined to lower‐body RE, it remained unclear whether the observed stability of MCAv reflected a training‐specific adaptation or a more general feature of cerebrovascular regulation during dynamic RE across different exercise modalities. To address this, MCAv responses were compared between lower‐ and upper‐body unilateral RE in untrained individuals (Korad et al., 2025b). If systemic BP magnitude or recruited muscle mass were important determinants of cerebrovascular responses, divergent MCAv responses between modalities would be expected. However, despite clear differences in systemic BP responses between modalities, MCAv responses were strikingly similar.

These findings challenge simplistic pressure‐centric models of cerebrovascular control during RE. Instead, they support the notion that the cerebral circulation responds primarily to the pattern of haemodynamic stress, rather than to the specific origin of the perturbation. Our data show that, whether the haemodynamic challenge arises from large lower‐body muscles or smaller upper‐body muscle groups, the brain appears to engage common regulatory strategies to preserve perfusion.

Taken together, the consistent preservation of MCAv across training status, recovery states and exercise modalities is consistent with active dCA, whereby cerebrovascular resistance is adjusted to buffer fluctuations in arterial BP. Although MCAv varied with changes in BP, the attenuated magnitude of these responses suggests that autoregulatory mechanisms were engaged and limited the transmission of pressure perturbations to the cerebral circulation, consistent with the frequency‐dependent nature of dCA. From an applied perspective, these findings open new avenues for exercise prescription aimed at enhancing cerebrovascular health. Identifying the intensity, frequency and modality thresholds that optimize beneficial haemodynamic stimuli remains an important challenge. In particular, the large and repeated fluctuations in perfusion pressure associated with RE might provide haemodynamic signals capable of influencing vascular structure and function over time. Investigation of shear stress responses during RE represents one potential avenue for understanding how these haemodynamic perturbations might contribute to cerebrovascular adaptation. Factors such as the magnitude and frequency of BP oscillations, the muscle mass engaged and the exercise intensity might influence the haemodynamic stimulus and associated shear‐related forces experienced by the cerebral vasculature. However, this remains a hypothesis requiring direct mechanistic investigation. Existing training studies suggest that dynamic RE can induce changes in cerebrovascular resistance at rest (Thomas et al., 2021), but the mechanisms and dose–response relationships underlying these adaptations remain poorly defined.

Collectively, these findings provide an observational framework, from which testable hypotheses regarding cerebrovascular regulation during dynamic RE can be generated. Future work should examine how acute cerebrovascular responses to RE vary with higher RE intensities, greater reliance on the Valsalva manoeuvre or bilateral movements involving larger muscle mass, because these factors might alter the haemodynamic stimulus.

A key methodological consideration is that the studies used a transcranial Doppler probe to measure MCAv, which quantifies MCA blood velocity rather than volumetric flow. Thus, future studies incorporating measures of arterial diameter and/or absolute blood flow (e.g., duplex ultrasound) would strengthen inferences regarding CBF. Answering these questions and implementing the additional methods will advance understanding of how the brain tolerates extreme haemodynamic challenges and might inform safer, more effective RE interventions for both athletic and clinical populations.

One conclusion, however, is clear: the brain is capable of maintaining relatively stable MCAv during dynamic RE despite substantial haemodynamic perturbations. Rather than being destabilized by the pressures imposed by RE, the cerebral circulation appears capable of buffering these pressures effectively. In the appropriate dose, the very haemodynamic stresses imposed by dynamic RE might highlight the capacity of the brain to buffer extreme haemodynamic challenges.

## AUTHOR CONTRIBUTIONS

Stephanie Korad, Toby Mündel and Blake Perry, conceptualised and design the research. Stephanie Korad and Blake Perry were responsible for data collection. Stephanie Korad, Toby Mündel and Blake Perry were responsible for data analysis, interpretation and drafting of the article. All authors have read and reviewed the article, provided critical feedback, and approved the final version of this manuscript. Furthermore, all authors agree to be accountable for all aspects of the work in ensuring that questions related to the accuracy or integrity of any part of the work are appropriately investigated and resolved. All persons designated as authors qualify for authorship, and all those who qualify for authorship are listed.

## CONFLICT OF INTEREST

None declared.
